# Chemically Induced Oncogenesis in the Peripheral Nervous System Is Suppressed in Congenic BDIX.BDIV-*Mss1* and -*Mss7* Rats

**DOI:** 10.1534/g3.115.021170

**Published:** 2015-11-02

**Authors:** Bernd Koelsch, Linda van den Berg, Christine Fischer, Bettina Winzen-Reichert, Andrea Kutritz, Andrea Kindler-Röhrborn

**Affiliations:** * Institute of Pathology, University Hospital of Essen, University of Duisburg-Essen, Essen, Germany; † Institute of Human Genetics, University of Heidelberg, Heidelberg, Germany

**Keywords:** cancer risk, malignant schwannoma, rodent, sex difference

## Abstract

Human malignant peripheral nerve sheath tumors (MPNSTs) are highly aggressive soft-tissue sarcomas with a poor prognosis that arise either in the context of neurofibromatosis 1 or sporadically. Inbred BDIX and BDIV rat strains highly susceptible and resistant, respectively, to the development of ethylnitrosourea-induced MPNST enable us to identify, by using methods not applicable in humans, variant alleles involved in the pathways underlying individual MPNST risk. On the basis of a genome-wide association analysis using reciprocal intercrosses of BDIX and BDIV, BDIV alleles of two loci on chromosome 10, *Mss1* and *Mss7*, were predicted to lower the risk of MPNST, the latter locus with a female bias. In this study we confirm the two nonoverlapping loci by exposing two congenic strains, BDIX.BDIV-*Mss1* (Mss1) and BDIX.BDIV-*Mss7* (Mss7), each carrying a BDIV genomic segment spanning the respective locus, to ethylnitrosourea. Compared with BDIX rats, the rate of MPNST is reduced 6.2-fold and 2.0-fold for Mss1 and Mss7 rats of both sexes, respectively. Although a moderate gain of survival time (30−50 days) is seen in Mss1 rats of both sexes and Mss7 males, Mss7 females survive 134 days longer than BDIX females. BDIV alleles at *Mss7* obviously cause a markedly increased intrastrain sex difference regarding survival time in Mss7 compared with BDIX rats. Fine mapping will lead to the identification of allelic variants modulating rat MPNST risk and subsequently to their human counterparts. This is of particular relevance, because so far neither gene nor anonymous sequence variants have been identified that influence the risk of human sporadic Schwann cell malignancy.

The risk of cancer in humans can be regarded as a complex genetic trait that is controlled by intricate interactions of susceptibility- and resistance-mediating gene variants with exogenic factors. Both determinants represent potential targets for cancer prevention. Malignant peripheral nerve sheath tumors (MPNSTs) are highly aggressive cancers with a poor prognosis; therefore, the development of targeted prevention measures is of great importance. Approximately 50% of all MPNSTs arise from neurofibromas, mostly of the plexiform type, in patients suffering from neurofibromatosis type 1 (NF1). Affected individuals carry germline mutations in the *NF1* gene that predispose them to various neural neoplasms. Sporadic MPNST arise *de novo* from peripheral nerves and frequently exhibit somatic mutations and losses of heterozygosity of the *NF1* gene. These patients do not carry a germline mutation in known tumor suppressor genes (Kolberg *et al.* 2013). The genetic risk of developing sporadic MPNST could be attributable to multiple common alleles with a low penetrance interacting with each other and/or environmental factors.

To identify allelic variants modifying MPNST risk, inbred rodent strains displaying differential tumor susceptibility are useful, because living conditions can be controlled, minimizing individual exposure to extrinsic factors. The genetic background of each strain is homozygous, representing a significant advantage over the genetic heterogeneity found in humans.

Quantitative trait loci influencing cancer risk can be identified by genome-wide association studies (GWAS) that use F_2_ intercrosses. Subsequently, the impact of each locus can be confirmed and quantified by induction of carcinogenesis in congenic rodent strains that carry on a susceptible genetic background homozygous resistance alleles at the locus in question. Subcongenic strains with progressively shorter resistance-mediating inserts finally allow the identification of the causative genes and eventually their human counterparts.

BD rat strains exhibit phenotypic differences regarding ethylnitrosourea (ENU)-induced carcinogenesis in the peripheral nervous system (Druckrey 1971). Although BDIX rats are highly susceptible to the development of MPNST in their trigeminal nerves, closely related BDIV rats display a very low risk that appears to be attributable to spontaneous cancer remission (Kindler-Rohrborn *et al.* 2000). Premalignant Schwann cells carrying a point mutation in the *Neu/Erbb2* gene diagnostic for the resulting MPNST emerge shortly after ENU exposure in both strains; however, mutant cells expand in trigeminal nerves of BDIX rats until full-blown tumors are present but disappear in BDIV nerve tissue about 3 months after neonatal ENU exposure (Kindler-Rohrborn *et al.* 2000). The immune system seems to be involved in this process, as demonstrated by T-cell−depleted BDIV rats that partially lose resistance toward MPNST development (Marx *et al.* 2009).

GWAS performed with F_2_ crosses of susceptible BDIX and resistant BDIV rats have been used to predict gene loci (*Mss1-Mss7*) mediating and counteracting, respectively, spontaneous cancer regression. Recently, *Mss4* locus BDIV alleles that reduce the risk of MPNST and increase survival time preferentially in females have been verified, narrowed down, and dissected into two subloci influencing MPNST resistance and/or enhancing its sex bias (Koelsch *et al.* 2011; van den Berg *et al.* 2015). Because BDIV alleles at the *Mss4* locus did not suffice to mediate tumor resistance to a similar extent as seen in BDIV rats, it became clear that further genes must be involved in cancer resistance of BDIV rats. In this study, we functionally confirm the effects of the nonoverlapping loci, *Mss1* and *Mss7*, located on chromosome 10, with the help of two congenic BDIX rat strains. These strains carry approximately 70 Mb and 23 Mb, respectively, long congenic BDIV-derived fragments spanning the corresponding loci that both exert profound effects on MPNST incidence and survival time compared with the parental BDIX strain.

## Materials and Methods

### Congenic breeding and genotyping

The inbred rat strains, BDIX (tumor-susceptible) and BDIV (tumor-resistant), were bred and maintained in the specific pathogen-free Central Animal Laboratory at the University Hospital of Essen, University of Duisburg-Essen. Congenic rat strains BDIX.BDIV-*Mss1* and BDIX.BDIV-*Mss7* (hereafter called Mss1 and Mss7) carry homozygous segments of the BDIV chromosome 10 on a BDIX background that cover the corresponding loci but do not overlap. They were generated according to the “speed congenics” method described previously (Koelsch *et al.* 2011; Visscher 1999) This method uses animals with heterozygous alleles at the chromosomal regions of interest but the greatest rate of homozygous BDIX alleles on all other chromosomes for backcrossing and requires microsatellite analysis of each generation. Both strains were derived from different N_3_ males bred from a BDIX × (BDIX × BDIV) N_2_ generation by additional backcrossing to BDIX males. In the N_6_ generation, potential founders underwent a whole-genome scan with 149 polymorphic microsatellite markers, 20 Mb apart on average, to detect residual BDIV alleles on the remaining chromosomes (Supporting Information, Table S1). With no heterozygous alleles detectable except in the congenic fragments, the congenic rat strains Mss1 and Mss7 were generated by brother−sister mating (Koelsch *et al.* 2011). Strains are available on request.

### Induction of peripheral nervous system tumors by ENU exposure

All experiments were performed according to the guidelines of “Gesellschaft für Versuchstierkunde/Society of Laboratory Animal Science” and the “Federation of European Laboratory Animal Science Associations.” Congenic strains Mss1 and Mss7 as well as BDIX rats of both sexes received a single subcutaneous injection of ENU (80 µg/g body weight) on postnatal day 1. All animals received treatment with a single lot of ENU to avoid variations of the concentration of the active substance. Because the incidence and latency time of MPNST for BDIX rats have been recorded many times, this strain could serve as quality control for the lot of ENU used. The animals were then observed for neurological symptoms on a daily basis. Animals displaying the slightest visible signs of tumor development, such as beginning cachexia, shortness of breath, paralysis, or behavioral abnormalities, were killed with isoflurane to minimize their suffering. Complete gross necropsy was performed. Animals were phenotyped with respect to the occurrence of trigeminal MPNST, other tumors, and to survival time. According to histopathologic analyses on a large cohort, ENU-induced trigeminal tumors invariably turned out to be MPNST. Therefore, trigeminal tumors could be diagnosed on the basis of their location, their macroscopic appearance, and their inseparability from the trigeminal nerve (Koelsch *et al.* 2013).

The study was approved by the local administration´s Ethical Committee on Experimental Animals (Landesamt für Natur, Umwelt und Verbraucherschutz, Recklinghausen, Germany; Reference Nr.84-02.04.2011A355), in accordance with national legislation.

### Construction of a polymorphism map of chromosome 10

Single-nucleotide polymorphism (SNP) data of the parental rat strains BDIX and BDIV were generated by the STAR Consortium (STAR Consortium *et al.* 2008). The SNP map of chromosome 10 was constructed by SNPlotyper (http://snplotyper.mcw.edu/) by the use of 1075 SNPs with unambiguous results for BDIX and BDIV rat strains. All positional data presented in this paper refer to the Baylor Map (Version 3.4/rn4 Nov. 2004; http://genome.ucsc.edu/cgi-bin/hgGateway)

### Statistical analysis

We analyzed times until visible signs of tumor development occurred to describe differences in latency times in different strains. As animals died or had to be killed within a few days after diagnosis, latency and survival time are almost identical. Rats lost as the result of nonmalignant disease or malignancies different from MPNST were considered “censored events.” Data are presented as Kaplan-Meier survival curves and on this basis median survival times and MPNST incidences on day 200 with 95% confidence intervals, respectively, were calculated. Strain- and sex-specific curves were compared with log rank tests. *P* < 0.05 was considered significant. Statistical evaluations were carried out with SigmaPlot 11 and SPSS V21.

### Data availability

Rat strains are available upon request. File S1 contains a list of microsatellites used in speed congenic breeding and final genome scan. 

## Results

### Genomic characterization of the congenic BDIX strains Mss1 and Mss7

**Figure 1:**
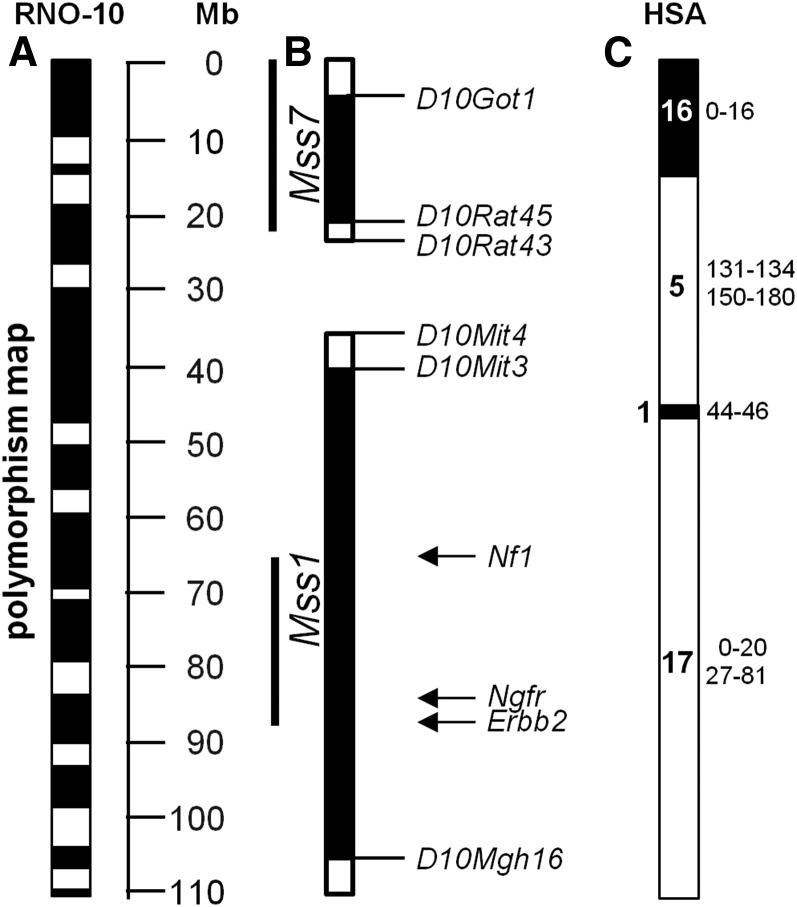
Physical map of rat chromosome 10 and alignment of congenic fragments Mss1 and Mss7. (A) White boxes indicate blocks of nonpolymorphic sequence >2 Mb between parental BDIX and BDIV rat strains inherited by a common ancestor; black boxes represent polymorphic segments. (B) Congenic BDIV fragments with flanking microsatellite markers shown as solid bars flanked by open bars representing the putative region of recombination. Thin lines indicate regions significantly associated with MPNST resistance mediated by BDIV alleles in a genome wide association analysis. (C) Homology map of rat chromosome 10 (RNO) and human chromosomes (HSA) indicating possible locations in the human genome harboring loci that influence MPNST risk. Human chromosomal fragments are indicated by bold digits for chromosome numbers. Lateral numbers mark their position on the corresponding chromosome in Mb.

The congenic BDIX strains Mss1 and Mss7, carrying BDIV-derived chromosome 10 segments that span the corresponding gene loci, were generated as described in the *Materials and Methods* section. Figure 1 shows the location of *Mss1* and *Mss7* congenic BDIV fragments on chromosome 10, the regions of polymorphic sequence between the two strains, as well as the orthologous human segment. The *Mss1* congenic fragment encompasses approximately the distal 75 Mb of chromosome 10 and harbors a maximum of 1511 genes according to the National Center for Biotechnology Information Map Viewer (http://www.ncbi.nlm.nih.gov/mapview), whereas the *Mss7* congenic fragment covers the proximal 23 Mb of chromosome 10. It contains 375 known genes at maximum.

### MPNST incidence and survival time analysis of *Mss1* and *Mss7* congenic strains compared with BDIX rats

To assess the MPNST susceptibility of congenic rat strains Mss1 and Mss7 relative to the parental BDIX strain 75 Mss1 rats (37 females and 38 males), 77 Mss7 rats (40 females and 37 males) and 109 BDIX rats (52 females and 57 males) were treated with ENU on postnatal day 1.

**Figure 2:**
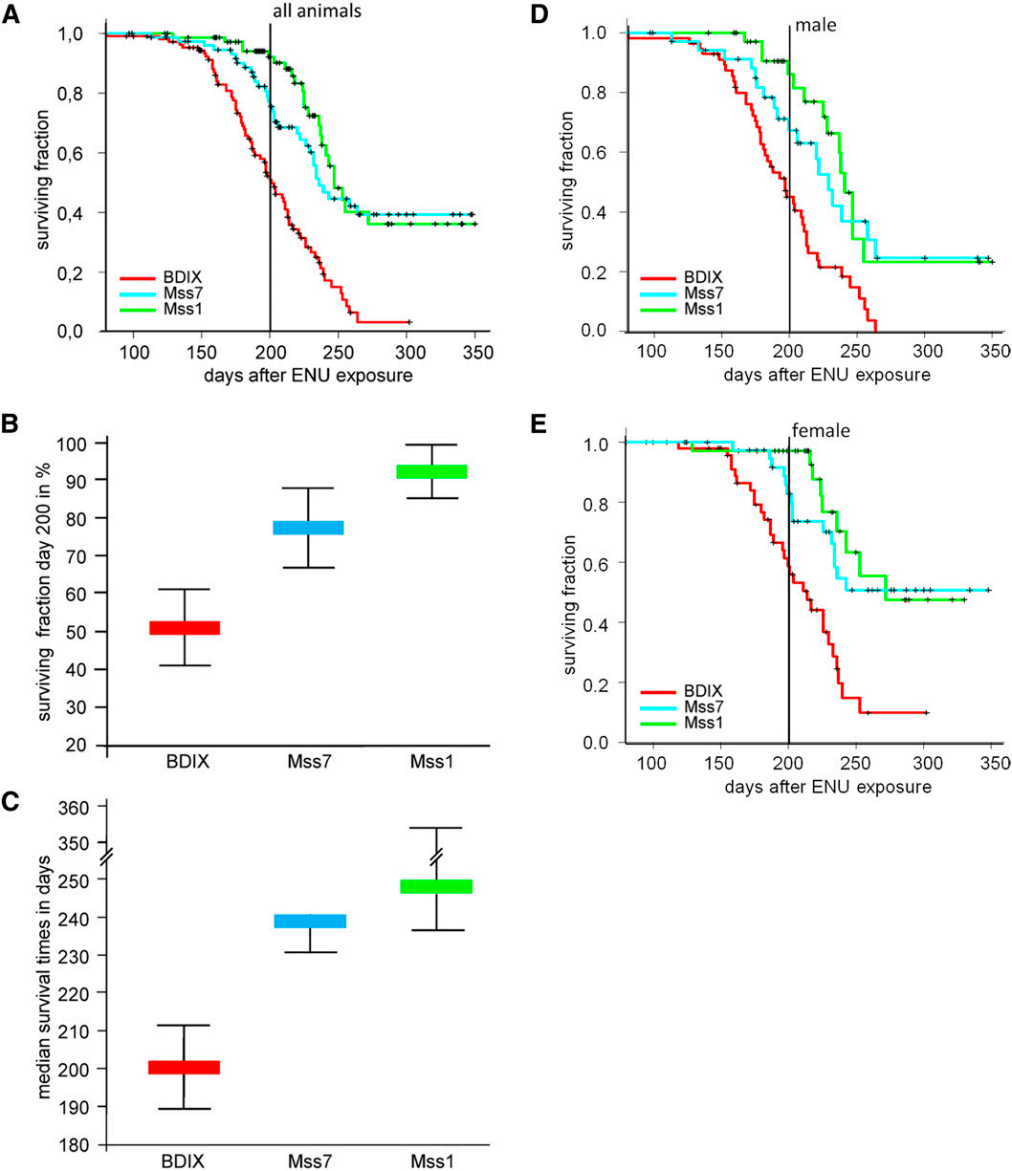
Comparison of the effect of different congenic BDIV segments introgressed into the BDIX genome on the risk of malignant peripheral nerve sheath tumor (MPNST) development. (A) Kaplan-Meier estimates of the distribution of times until death or euthanasia of BDIX, Mss7, and Mss1 rats attributable to MPNSTs induced by ethylnitrosourea (ENU) on postnatal day 1. Animals that died or were killed because of tumors other than MPNSTs or other diseases were counted as censored observation; their survival times are marked with ticks. (B) Effect size of each congenic segment on the MPNST incidence on day 200 after ENU exposure and (C) on median survival times. Thin lines show 95% confidence intervals. (D) and (E) Sex-separated Kaplan-Meier estimates of distributions of times until death or euthanasia of male and female BDIX, Mss7, and Mss1 rats.

Kaplan-Meier estimates of survival time distributions were performed for each rat strain. The distributions of survival times of all Mss1 and all Mss7 rats, respectively, showed highly significant differences to the Kaplan-Meier curve of BDIX rats as judged from the log rank test (*P* = 1.5 × 10^−9^ and 2.8 × 10^−6^, respectively; see Figure 2A). Although the surviving fraction of BDIX rats shows a continuous decline attributable to the occurrence of MPNST between 140 and 260 days, there was a later onset of the decline in Mss1 and Mss7 rats, but the same endpoint as in BDIX rats. Only one MPNST was detected in animals of all three strains older than 260 days (N = 25). The Kaplan-Meier−based estimates of median survival times of 247 and 239 days, respectively, and MPNST incidences on day 200 after tumor induction of 7.9% and 22.5%, respectively, were calculated for congenic Mss1 and Mss7 animals of both sexes. BDIX rats exhibited a median survival time of 201 days and a MPNST incidence of 49% (Table1; Figure 2B). The distinct confidence intervals of median survival times and MPNST incidences for Mss1 and Mss7 related to the BDIX values underscored their significance (Figure 1C). Accordingly, Mss1 and Mss7 rats gained 46 and 38 days of lifetime, respectively, whereas their MPNST risk on day 200 after ENU exposure was 6.2- and 2.0-fold reduced compared with BDIX rats (Table 1).

**Table 1:** Comparative survival times and tumor incidences of BDIX, Mss1, Mss7, and BDIV rats of both sexes after ENU exposure on postnatal day 1 based on Kaplan-Meier estimations

	*BDIX*			*Mss1*			*Mss7*		
	♂+♀	♂	♀	♂+♀	♂	♀	♂+♀	♂	♀
	n = 109	n = 57	n = 52	n = 75	n = 38	n = 37	n = 77	n = 37	n = 40
Median survival time, days	201	197	214	247	241	272	239	229	>348
95% confidence interval, days	188/213	179/210	189/230	237/355	225/255	236/-	229/-	200/264	232/-
Gain in survival time *vs.* BDIX, days	−	−	−	46	44	58	38	32	>134
MPNST incidence on day 200, %	49.0	55.0	41.5	7.9	13.9	2.9	22.5	32.7	17.2
95% confidence interval, % low/high	39/59	41/69	26/57	1/15	1/27	0/8	12/33	16/50	5/30
Fold lower MPNST risk *vs.* BDIX on day 200	−	−	−	6.2 ×	4.0 ×	14.3 ×	2.0 ×	1.7 ×	2.4 ×

ENU, ethylnitrosourea; MPNST, malignant peripheral nerve sheath tumor.

The differences of sex-separated Kaplan-Meier survival curves for Mss1 and Mss7 rats compared with their BDIX counterparts also were highly significant (males: Mss1 and Mss7 *vs.* BDIX, respectively; *P* = 0,00013 and 0,005, respectively; females: Mss1 and Mss7 *vs.* BDIX, respectively ; *P* = 0,000044 and 0,00093, respectively; Figure 2, D and E). Female Mss1 and Mss7 rats gained 58 and >134 days, respectively, of median survival time through the integration of the respective BDIV fragments on chromosome 10, whereas congenic Mss1 and Mss7 males lived 44 and 32 days, respectively, longer than their male BDIX counterparts (Table 1).

**Figure 3:**
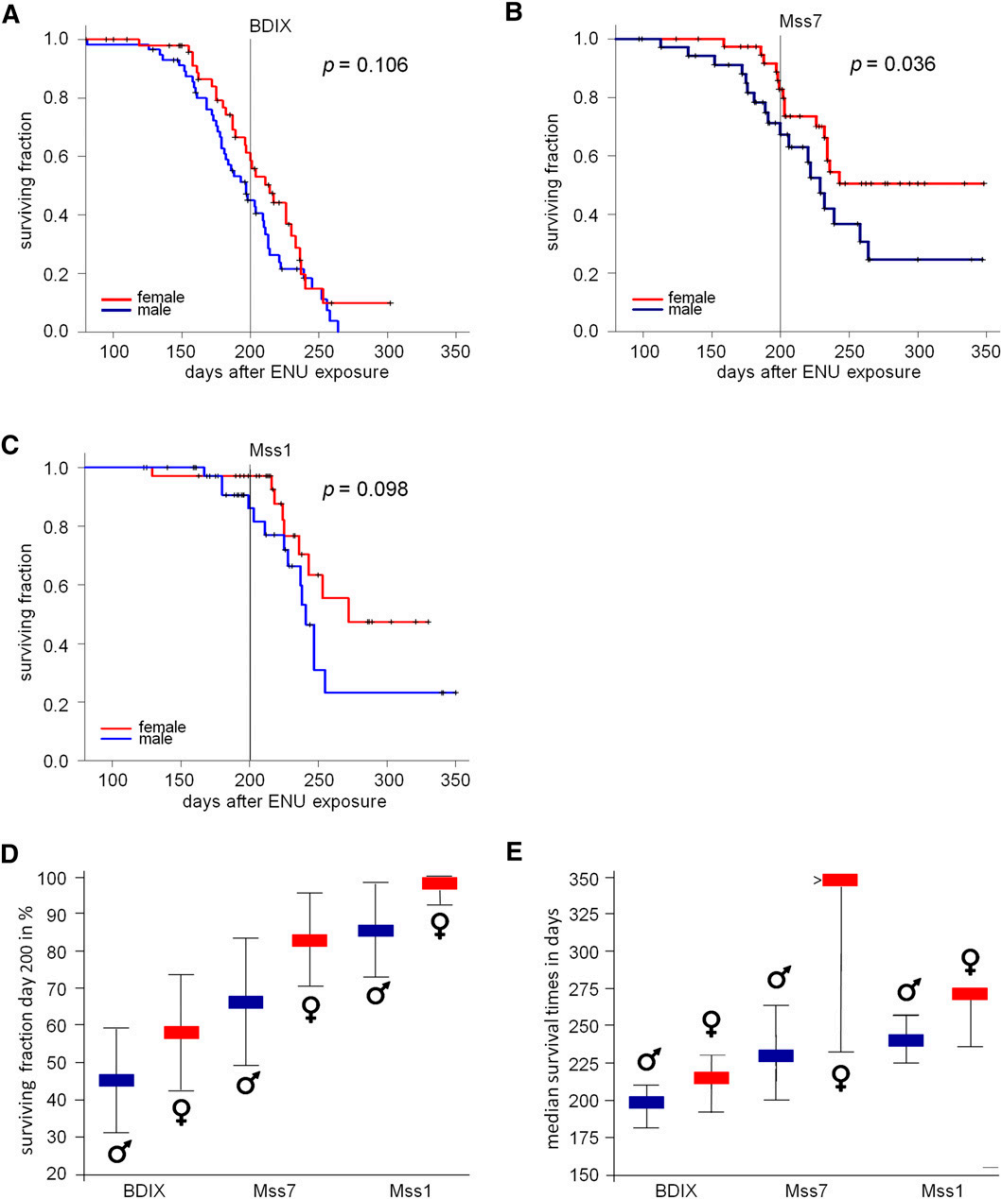
Sex-dependent, intrastrain effects of congenic BDIV segments on MPNST incidence and survival time. Sex-separated Kaplan-Meier estimates of the distributions of times until death for (A) BDIX females and males; (B) Mss7 females and males; and (C) Mss1 females and males. (D) Effect size of each congenic segment on MPNST incidence at day 200 and (E) effect size on median survival times. Thin lines represent 95% confidence intervals.

Female Mss1 and Mss7 rats exhibited a tumor incidence of 2.9% and 17.2% on day 200 after ENU exposure compared with 41.5% in BDIX females, which was consistent with a 14-fold and a twofold reduction of relative tumor risk (Table 1; Figure 3, A−C). A total of 13.9% of male Mss1 and 32.7% of male Mss7 rats developed MPNST, whereas male BDIX rats exhibited an incidence of 55.0%, so that the reduction of tumor risk amounted to 4- and 1.7-fold, respectively. Nevertheless, in Mss1 and Mss7 rats, intrastrain sex differences regarding the incidence of MPNST did not surpass the difference recorded for BDIX males and females (see Figure 3, A−C). Medians of MPNST incidences between Mss1 males and females and Mss7 males and females differed by 11% and 15%, respectively, whereas we recorded a difference of 14% in BDIX rats, in all cases in favor of females (Figure 3D). The overlapping confidence intervals of values for males and females may reflect a limited significance of the sex differences observed in each strain (Figure 3D). In contrast, the sex difference in median survival time of 17 days in BDIX rats, again in favor of females, was exceeded by 14 days in *Mss1*, and > 102 days in Mss7 rats (see Figure 3E; distinct confidence intervals between Mss7 values related to BDIX and Mss1 values).

## Discussion

Congenic rodent strains generally are used to confirm gene loci previously predicted by GWAS using F_2_ animals and to estimate the effect size of a distinct locus on the phenotype in question. Nevertheless, effect sizes of loci can vary widely between F_2_ intercross- and congenic animals. This finding is partly attributable to the heterogeneous genetic background of F_2_ rats, allowing multiple interactions with sequence variants of both strains, whereas in congenic rats the recipient alleles only are present beside the donor insert. In extreme cases this can lead to full penetrance of the locus or to a complete suppression of the effect, which is hard to distinguish from the absence of the causal gene(s) in the congenic fragment. It is therefore useful to start congenic breeding with a large donor fragment, although this may contain additional loci influencing the phenotype.

By ENU exposure of congenic Mss1 and Mss7 rats carrying homozygous BDIV alleles at the corresponding loci on chromosome 10, we were able to confirm and quantify the tumor resistance effects of gene variants and/or variant regulatory elements located in these segments (Figure 1B). Compared with BDIX rats, Mss1 and Mss7 animals showed markedly increased survival times and lower tumor rates.

The *Mss1* locus had been detected by a targeted linkage analysis with chromosome 10 simple sequence length polymorphisms after the discovery that ENU-induced MPNST of (BDIV × BDIX) F_1_ rats had been shown previously to display losses of heterozygosity on this chromosome usually losing the BDIV resistance allele. This pointed to the existence of a BDIV rat strain−specific negative regulatory element possibly mediating MPNST resistance. The linkage core region was located around *D10Wox23* at 84.7 Mb (Kindler-Rohrborn *et al.* 1999).

As expected, the homozygous 75 Mb BDIV fragment reaching approximately from 35 Mb to 110 Mb on chromosome 10 of Mss1 rats had a strong effect on Schwann cell oncogenesis. The courses of Kaplan-Meier curves of ENU-exposed BDIX and Mss1 rats run in parallel for 150 days; however, tumor development is delayed in Mss1 rats, and is no more seen in animals older than 260 days (Figure 2A). Therefore, the development of MPNST does not seem to be postponed but restricted to a certain time window. Consequently, the tumor rate 200 days after ENU exposure dropped from 49% in BDIX rats to 7.9% in congenic Mss1 rats (Figure 2B). Additionally, the survival time of Mss1 rats was 46 days longer compared with BDIX rats, which corresponds to approximately 5 yr of the human life span.

The MPNST risk on day 200 of female Mss1 rats was 14-fold reduced and the survival time >8 weeks longer compared with BDIX females, whereas male Mss1 rats showed a fourfold reduction of tumor incidence and a >6 weeks longer survival time compared with BDIX males (Figure 3, D and E). Nevertheless, neither the intrastrain sex bias of MPNST incidence nor of the survival time in congenic Mss1 rats significantly exceeded the differences observed in female and male BDIX rats. Therefore, the BDIV fragment introgressed in Mss1 rats does not seem to harbor genetic factors influencing sex-dependent tumor risk as recently described for the *Mss4* locus (Koelsch *et al.* 2011; van den Berg *et al.* 2015). This finding is consistent with the results obtained from reanalyses of linkage data considering both sexes separately (Koelsch *et al.* 2006). Given the fact that the BDIV *Mss1* fragment integrated in the genome is about 75 Mb long and contains 1500 genes, candidates can only be suggested on the basis of gene function at this point (Figure 1B). It is not likely, however, that the BDIV allele of the *Nf1* gene—responsible for a large portion of human MPNST—located at 65 Mb on rat chromosome 10 causes the *Mss1* resistance effect, as this gene previously did not show association to MPNST incidence and survival time (Roth *et al.* 2008). It is tempting to speculate that the *Ngfr* gene (at 84 Mb) is an interesting candidate. *Ngfr*, crucial for Schwann cell development, is expressed transiently in these cells and has been shown to be a central regulator of glioma invasion (Bentley and Lee 2000; Johnston *et al.* 2007). During ENU-induced oncogenesis in the trigeminal nerves of BD rats Ngfr protein was detected in proliferating *Erbb2* mutant premalignant Schwann cells as well as in the resulting MPNST (S. Levin and A. Kindler-Röhrborn, unpublished data). A fraction of human MPNST also shows Ngfr expression (Olsen *et al.* 2006). Interestingly, its ligand, nerve growth factor (Ngf), has been demonstrated to influence ENU-induced rat and mouse MPNST frequency. Treatment with exogenous Ngf caused reduction of trigeminal MPNST frequency, while injection of antibodies directed against Ngf reduced serum levels of the bioactive protein resulting in an increased MPNST incidence (Raju *et al.* 1989; Vinores and Perez-Polo 1980).

The BDIV fragment covering the *Mss7* locus in Mss7 rats is located 15 Mb upstream of the congenic *Mss1* interval (Figure 1). It confines the restriction of tumor development to a similar time span as seen in Mss1 rats. The *Mss7* locus was shown to mediate a female-biased MPNST resistance effect in (BDIV × BDIX) F_2_ rats (Winzen *et al.* 2006). However, as previously seen for the *Mss4* locus, both male and female Mss7 congenic rats did benefit from homozygous BDIV *Mss7* alleles, though females to a markedly greater extent (Koelsch *et al.* 2011). Compared with BDIX rats of the same sex, there is a pronounced female-biased effect on survival time (>134 additional days in females *vs.* 32 days for male rats), whereas the reduction of MPNST incidence on day 200 after ENU exposure is about the same in both sexes (2.4-fold and 1.7-fold reduced risk for Mss7 females and males, respectively). Accordingly, the Mss7 intrastrain sex difference regarding MPNST incidence on day 200 does not surpass the one observed in BDIX rats, whereas the marked sex bias in survival time seen in Mss7 rats leads to a sex difference by far exceeding the one observed in female and male BDIX rats (Figure 3D). These results clearly indicate the presence of at least one gene variant and/or regulatory element located in the BDIV fragment introgressed which are involved in resistance mechanisms against ENU-induced MPNST development. BDIV alleles at the *Mss4* locus are known to be involved in female preference of MPNST resistance (van den Berg *et al.* 2015). Therefore, the gene or regulatory element underlying the effect of the *Mss7* locus should simultaneously affect MPNST resistance and female preference. Alternatively, *Mss7* may be a compound locus, with several genetic factors controlling these processes. The sex-bias of cancer resistance in favor of female congenic Mss7 rats might be effected by strain-specific alleles of SNPs in regulatory regions of candidate genes creating binding sites for hormone-dependent transcription factors or by the genome-wide expressional fingerprint of candidate hormone−dependent transcription factors residing in this locus.

In the future, both loci have to be narrowed down by generating subcongenic strains through further backcrossing and exposing them to ENU. Specially, the regions polymorphic between BDIX and BDIV rat strains, which are likely to harbor the underlying genes, have to be considered (Figure 1A). Whereas in the case of *Mss1* a candidate gene approach appears promising, the Mss7 congenic segment does not harbor obvious functional candidate genes that might be involved in spontaneous remission of MPNST as observed in BDIV rats. Nevertheless, comparative sex-specific expression analyses via use of the trigeminal nerves of BDIX and Mss7 rats also should help to unveil genes in *Mss7* contributing to sex-biased MPNST resistance as we could recently show for the *Mss4* locus (van den Berg *et al.* 2015). Because cancer risk-modifying genes do not necessarily have to be expressed in the tissue the tumor originates from expression profiles also should be recorded from other organs that are likely to be involved, such as hormone glands as well as the immune system, which shows a strong sexual dimorphism (Smits *et al.* 2011).

Because approximately 50% of human MPNSTs arise “spontaneously” meaning unlinked to a known cancer predisposing gene the two loci confirmed here might harbor gene variants responsible for a fraction of the non-*NF1*−linked human MPNST cases. Should the same genes that cause the MPNST-protective effects of *Mss1* or *Mss7* loci also be involved in human MPNST development, these genes will be most probably located in regions of human chromosomes 1, 5, 16, and 17, representing the human counterparts of rat BDIV congenic fragments (Figure 1C). In the future, Mss1 and Mss7 alleles of both rat strains should be comparatively analyzed by next-generation sequencing to identify polymorphisms in coding regions and in the noncoding sequence. Additionally, gene expression profiling should be performed to identify differently expressed genes located in the loci as well as the transregulatory fingerprint BDIV and BDIX alleles at *Mss1* and *Mss7* might induce.

At this point, *Mss1*, *Mss4*, and *Mss7* are the first functionally confirmed gene loci influencing the risk of Schwann cell neoplasia, other than *NF1*, *NF2*, and *SMARCB1*.

## 
